# THE ROLE OF PERSON- AND FAMILY-ORIENTED LONG-TERM SERVICES AND SUPPORTS

**DOI:** 10.1093/geroni/igad104.2036

**Published:** 2023-12-21

**Authors:** Chanee Fabius, Safiyyah Okoye, Mingche Wu, Andrew Jopson, Julia Burdgorf, Jeromie Ballreich, Daniel Scerpella, Jennifer Wolff

**Affiliations:** Johns Hopkins University, Baltimore, Maryland, United States; College of Nursing and Health Professions, Drexel University, Philadelphia, Pennsylvania, United States; Johns Hopkins University, Baltimore, Maryland, United States; Johns Hopkins University, Baltimore, Maryland, United States; Center for Home Care Policy & Research, New York, New York, United States; Johns Hopkins Bloomberg School of public health, Baltimore, Maryland, United States; Johns Hopkins Bloomberg School of Public Health, Baltimore, Maryland, United States; Johns Hopkins University, Baltimore, Maryland, United States

## Abstract

Long-term services and supports (LTSS) in the United States are defined by its patchwork and unequal nature. Data constraints obscure our understanding of inequities in LTSS care experiences and factors that may attenuate them. We advance a conceptual framework of LTSS-relevant environmental domains. We then link measures from public use datasets to person-reported care experiences from the 2015 National Health and Aging Trends Study. We assess relationships between domains of LTSS-relevant environmental characteristics and three LTSS care experiences: 1) adverse consequences due to unmet need, 2) participation restrictions in valued activities, and 3) subjective well-being. We examine 2,411 older adults with disabilities, and key subgroups by race, dementia, and Medicaid enrollment status. We find that for the roughly 10.4 million older adults with disabilities in the community, the LTSS environment is associated with care experiences, but the direction of the relationship varies by domain. Measures of neighborhood social and economic deprivation (e.g., poverty, social cohesion) are associated with experiencing adverse consequences due to unmet needs. Measures of the health care and social services delivery environment (e.g., Medicaid Home and Community-Based Service Generosity, average direct care worker wage, paid family leave) are inversely associated with experiencing participation restrictions. Select measures of the built and natural environment (e.g., housing affordability) are associated with participation restrictions and low subjective well-being. Relationships between measures of LTSS-relevant environmental characteristics and care experiences generally held in directionality but were attenuated for key subpopulations. Results reinforce the importance of strengthening environmental policies that support aging in place.

